# An Unusual Association between Unilateral Intracranial Vessels Occlusion with Iron Deficiency Anaemia and Alpha-Thalassemia Trait: A Case Report

**DOI:** 10.1155/2011/271560

**Published:** 2011-12-20

**Authors:** Yeong Yeh Lee, Shalini Bhaskar

**Affiliations:** Department of Medicine, School of Medical Sciences, Universiti Sains Malaysia, Kubang Kerian, Kelantan, 16150 Kota Bharu, Malaysia

## Abstract

We report a 33-year-old Malay woman presented with acute left dense hemiparesis and an NIHSS score of 11/15. Computed tomography (CT) scan brain showed a massive right middle cerebral artery (MCA) territory infarct. The right internal carotid artery (ICA) and right proximal MCA were shown occluded from digital substraction angiography (DSA). Carotid dissection, carotid canal anomaly, and intercavernous communication were systematically ruled out. She had no risk factors for atherosclerosis. The connective tissue screening and thrombophilic markers were negative. However, she was anaemic on admission and subsequent investigations revealed that she had alpha-thalassemia and iron deficiency anaemia. The right ICA remained occluded from a repeat CT cerebral angiogram after one year, but otherwise she was neurologically stable. This case illustrates an unusual association between intracranial vessel occlusion with iron deficiency anaemia and alpha-thalassemia trait.

## 1. Introduction

A young stroke patient required exhaustive investigations to rule out secondary causes and often the cause may not be apparent. We report an unusual association between a young stroke patient having unilateral intracranial vessels occlusion with iron deficiency anaemia and alpha-thalassemia trait. 

## 2. Case Presentation

A 33-year-old Malay lady para 4 + 2 without any previous medical history presented with acute onset of left-sided body weakness one day prior to admission in March 2006. There was a preceding history of vomiting but no headache, loss of consciousness, seizure, or fever. She did not have any difficulties in her speech, no blurring of vision, no difficulty in swallowing, no vertigo and gait imbalance. She denied any family history of stroke, valvular heart disease, blood disorders, or malignancies. However, she had history of two abortions both after her first trimester. She was not on any drugs including traditional medications. 

Physical examination revealed a fully conscious lady, following commands with no speech defects. Her pulse rate was 64/min and regular. Her supine blood pressure was 112/64 mmHg with evidence of postural hypotension. She appeared pale but not jaundiced. The cardiovascular, respiratory, and abdominal examinations were unremarkable. There was no neck stiffness, cranial nerve palsies, and no carotid bruit. She had a right dense hemiparesis with power of 0/5 and an NIHSS score of 11/15. She also exhibited a mild left-sided neglect syndrome and left hemianopsia. Other right hemispheric dysfunction includes a flat effect and confabulation. 

**Figure 1:**
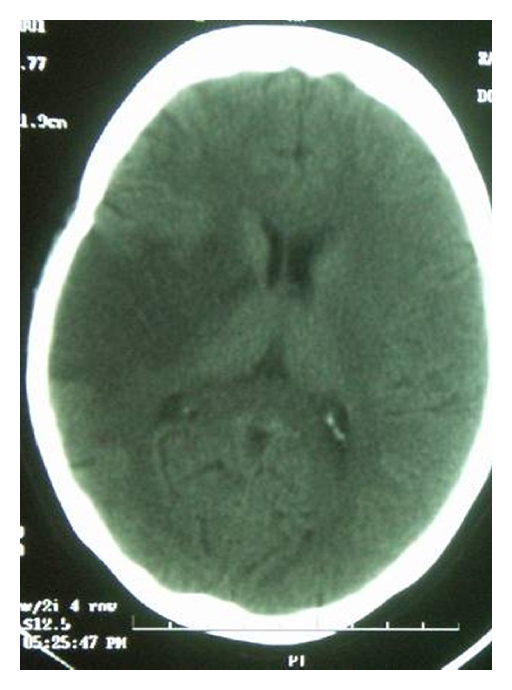
Plain CT scan brain showing a massive right MCA territory infarct during admission.

A plain computed tomography (CT) of brain on admission showed a massive infarct at the right middle cerebral artery (MCA) territory with a dense right MCA sign (Figure 1). The antinuclear antibody and the rheumatoid factor were negative. Antiphospholipid antibodies and coagulation profiles including prothrombin time and activated prothrombin time were normal. Other thrombophilic markers including homocysteine, protein C, protein S, and factor V Leiden mutations were all negative. The fasting sugar level was 4.2 mmol/L, and her total cholesterol was 4.58 mmol/L. Her full blood picture revealed a microcytic hypochromic picture and a hemoglobin level of 9.0 g/dL. A combination of haemoglobin electrophoresis and genetic testing confirmed that she had alpha-thalassemia trait. There was no evidence of sickle cell anaemia. She also had concomitant iron deficiency anaemia as a result of her poor nutrition after her childbirth a year ago. Her relatively poor income background, low education, and unemployment exacerbated her poor nutrition. Both the chest X-ray and the electrocardiogram (ECG) were normal. Transthoracic echocardiography (TTE) did not show any valvular lesions, thrombus, or vegetations. There was no evidence of a patent foramen ovale. 

**Figure 2:**
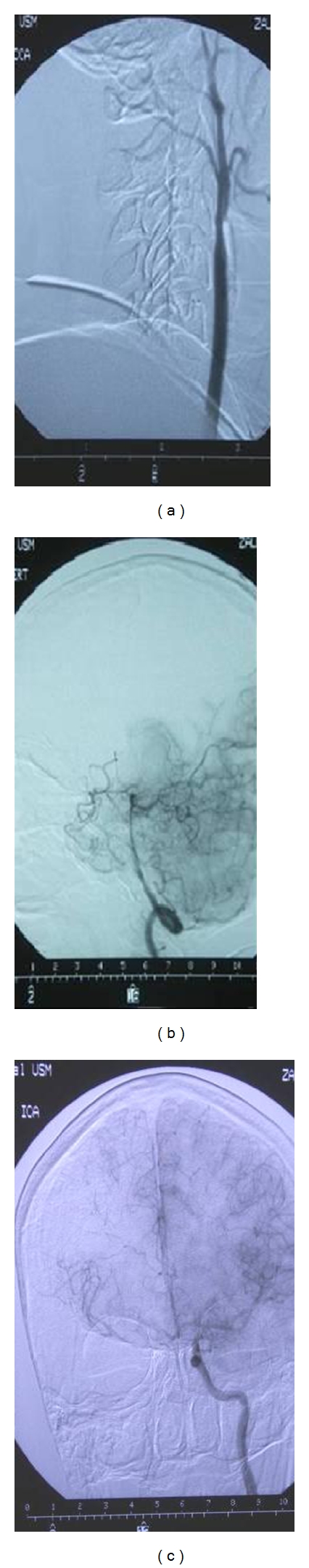
Digital subtraction angiography performed within 2 weeks of admission. The left-right common carotid artery lateral view was showing an absence of right internal carotid artery (ICA) branch (a). The middle-left vertebral artery lateral view was showing increased collaterals from posterior communicating artery (b). Finally the right-left ICA anterior view was showing a cross-supply of collaterals into the right ICA territory (c).

Within 2 weeks after her admission, an occluded right internal carotid artery (ICA), a small and a stenotic right MCA with no opacification of the terminal branches were shown in the digital subtraction angiography (DSA) (Figure 2). There were collaterals supplying the right parietal lobe from right posterior communicating artery (PCA) as well as cross-supply from the left ICA but no intercavernous communication. There was no evidence for carotid artery dissection, neither was there an absence of or a small carotid canal from the CT scan. 

We followed her progress over the next one year. There were no new neurological deficits since the index event, but she never regained full motor function despite regular physiotherapy. She complained of intermittent headache which was relieved with usual painkillers and rest. A CT cerebral angiography was repeated after one year in April 2007. The right MCA became patent but still relatively small. The right ICA remained occluded, and the arch aortogram did not reveal any anatomical variance. The cross-supply to the right cerebral hemisphere was provided by the left ICA and PCA. She was put on lifelong aspirin and perindopril with a view to start on another antiplatelet. 

## 3. Discussion

The frequency of intracranial internal carotid artery (ICA) occlusion in autopsy studies was ranging between 0 and 30% [1]. Intracranial ICA atherosclerosis and occlusion have unexplained tendencies to be more severe in Blacks and Asians. Some studies suggested a worst prognosis with intracranial occlusion than extracranial occlusion [2]. The majority of fatal occlusive thromboses of intracranial ICA are likely embolic in origin, and the source is not limited to the heart and aorta but also arises from the ipsilateral carotid sinus. We presented an idiopathic case of sudden occlusion of right intracranial internal carotid artery (ICA) and right middle cerebral artery (MCA). 

Strokes due to intracranial right MCA and right ICA occlusions were unusual but not uncommonly reported in the literature. Atherosclerosis of intracranial ICA commonly involved cavernous and supraclinoid segments, but more often the lesions were calcific and often not stenotic [1]. The traditional vascular risk factors including hypertension, hyperlipidemia, diabetes mellitus, coronary artery disease, or peripheral vascular disease were absent in this young patient. Therefore, thrombosis as a result of atherosclerosis as a cause of her vessel occlusion was unlikely. The absence of cardioembolic source and absence of a patent foramen ovale from transthoracic echocardiography exclude cardiac as a cause for vessel occlusion. Transoesophageal echocardiography may have been a better investigation, but unfortunately this test was not available in our centre at that time. Extracranial carotid artery (ECA) stenosis or occlusion was a potential source of emboli, but it was not seen on angiography. Carotid dissection can cause a similar presentation, but the patient did not complain of severe headache and there was no angiographic evidence of dissection.

Antiphospholipid syndrome and systemic lupus erythematosus (SLE) were other possible causes of stroke in view of her repeated abortions. However, her antiphospholipid antibodies and antinuclear antibody (ANA) were repeatedly negative. The normal coagulation profiles and thrombophilic markers effectively rule out procoagulant state as a cause. A developmental anomaly of her intracranial arteries can be a potential cause of the vessels occlusion where agenesis [3], aplasia, or hypoplasia of internal carotid [4] were reported in the available literature. However, developmental anomaly is usually characterized by the absence or a small carotid canal which was not demonstrated from the CT scan in this patient. Furthermore, there was no intercavernous communications [5] that existed between the left ICA and the right ICA. Moyamoya disease is another consideration; however, the presentation was not the usual hemorrhagic type, vessels involvement were not bilateral (though there were reported cases of unilateral involvement [6, 7]), and there was no typical sign of “puff of smoke.” 

Alpha-thalassemia trait on its own will not cause sufficient anaemia to result in stroke. The risk of stroke is reduced further if there is concomitant sickle cell anaemia [8, 9], but this was not present in this particular patient. There were no reported studies on association between alpha-thalassemia trait and stroke. However, iron deficiency anaemia has been associated with cerebrovascular disease [10, 11], but the actual underlying mechanism for the association is still largely speculative. Thrombocytosis associated with iron deficiency anaemia may lead to thrombus formation since platelet aggregation is required for thrombosis [10]. Severe anaemia may cause reversible focal hypoxic deficits unmasking underlying silent brain lesions including atherosclerotic disease and metabolic encephalopathy [12, 13]. The thrombus formation within the intracranial vessels may also be due to turbulence in flow which damage endothelium and promote platelet aggregation. It was observed that arterial bruits were common in severe anaemia and bruits are a physical sign for turbulence [14, 15]. 

While we may not eventually point out the actual cause for the intracranial vessels occlusion in this young stroke patient, but we find comfort that her progress over one year was stable. In a recent study on anaemia and outcome after acute stroke involving 859 patients, it was found that both low and high levels of haemoglobin predicted poor 1-year outcome [16]. 

## 4. Conclusion

Firstly, this case highlights an unusual association between intracranial vessel occlusion with iron deficiency anaemia and alpha-thalassemia trait. Secondly, it is to highlight the importance of investigating and exclusion of other causes of stroke in a young patient.
